# Supplementary material for: Considering the Value of 3D Cultures for Enhancing the Understanding of Adhesion, Proliferation, and Osteogenesis on Titanium Dental Implants
Source: Biomolecules. 2023 Jun 28;13(7):1048. doi: 10.3390/biom13071048 (PMC10377630; doi:10.3390/biom13071048)
Supplement: Supplementary file 1 [file biomolecules-13-01048-s001.zip › biomolecules-2407249-supplementary.pdf]

## Supplemental Tables

**Supplemental Table S1: Summary of the adhesion results on the different Titanium Implants surface.** The table shows the amount of counted cells and the relative statistical significance at the tested timepoints. The data is significant with $p \leq 0.001$ (ANOVA followed by Bonferroni's post hoc test or paired t-test). *24h-3days, ^24h-7days and %3-7days (paired t-test) indicating significance.

| *Reference Implant* | *Timepoints* | *Between Timepoints* | *Cell Number* | *Implant Type* | *24 hours* | *3 days* | *7 days* |
|---|---|---|---|---|---|---|---|
| ***Al Blast*** | | | | **Ti Blast** | 1 | 1 | 1 |
| | 24 hours | T1-T2(*) 0.784 | 65.3 ± 30 | **Laser Mach** | <0.001 | 0.126 | <0.001 |
| | 3 days | T1-T3(^) <0.001 | 67.3 ± 37.6 | **Laser Sint** | <0.001 | 0.002 | <0.001 |
| | 7 days | T2-T3(%) <0.001 | 35.7 ± 16.2 | **Cor Blast** | 1 | <0.001 | <0.001 |
| ***Cor Blast*** | | | | **Ti Blast** | 1 | 0.001 | 0.024 |
| | 24 hours | T1-T2(*) <0.001 | 59.2 ± 48 | **Al Blast** | 1 | <0.001 | <0.001 |
| | 3 days | T1-T3(^) 0.006 | 145 ± 71.7 | **Laser Mach** | <0.001 | 0.295 | 1 |
| | 7 days | T2-T3(%) 0.080 | 114 ± 64.2 | **Laser Sint** | <0.001 | 1 | <0.001 |
| ***Ti Blast*** | | | | **Al Blast** | 1 | 1 | 1 |
| | 24 hours | T1-T2(*) 0.550 | 87.1 ± 20.1 | **Laser Mach** | <0.001 | 0.889 | <0.001 |
| | 3 days | T1-T3(^) 0.011 | 79.2 ± 56.2 | **Laser Sint** | 0.001 | 0.032 | <0.001 |
| | 7 days | T2-T3(%) 0.278 | 62.7 ± 40.6 | **Cor Blast** | 1 | 0.001 | 0.024 |
| ***Laser Sint*** | | | | **Ti Blast** | 0.001 | 0.032 | <0.001 |
| | 24 hours | T1-T2(*) 0.084 | 152.8 ± 36.4 | **Al Blast** | <0.001 | 0.002 | <0.001 |
| | 3 days | T1-T3(^) 0.008 | 127.4 ± 62.14 | **Laser Mach** | 1 | 1 | 0.003 |
| | 7 days | T2-T3(%) 0.008 | 199.6 ± 63.2 | **Cor Blast** | <0.001 | 1 | <0.001 |
| ***Laser Mach*** | | | | **Ti Blast** | <0.001 | 0.889 | <0.001 |
| | 24 hours | T1-T2(*) <0.001 | 159 ± 32.8 | **Al Blast** | <0.001 | 0.126 | <0.001 |
| | 3 days | T1-T3(^) 0.162 | 108.5 ± 39.9 | **Laser Sint** | 1 | 1 | 0.003 |
| | 7 days | T2-T3(%) 0.143 | 138.2 ± 83 | **Cor Blast** | <0.001 | 0.295 | 1 |

**Supplemental Table S2: Summary of the viability results on the different Titanium Implants surface.** The table shows viability across the time-points respect the different implant types the relative statistical significance at the tested timepoints. The data is significant with $p \leq 0.001$ (ANOVA followed by Bonferroni's post hoc test or paired t-test). *24h-3days, ^24h-7days and %3-7days (paired t-test) indicating significance.

| *Reference Implant* | *Timepoints* | *Between Timepoints* | *Viability* | *Implant Type* | *24 hours* | *3 days* | *7 days* |
|---|---|---|---|---|---|---|---|
| ***Al Blast*** | | | | **Ti Blast** | 1 | 1 | 0.139 |
| | 24 hours | T1(*) 0.016 | 0.03 ± 0.56 | **Laser Mach** | 1 | 0.008 | 0.007 |
| | 3 days | T2(^) 0.026 | -0.5 ± 0.53 | **Laser Sint** | 1 | <0.001 | <0.001 |
| | 7 days | T3(%) <0.001 | -0.88 ± 0.83 | **Cor Blast** | <0.001 | 1 | <0.001 |
| ***Cor Blast*** | | | | **Ti Blast** | <0.001 | 1 | <0.001 |
| | 24 hours | T1(*) <0.001 | 1.7 ± 1.66 | **Al Blast** | <0.001 | 1 | <0.001 |
| | 3 days | T2(^) <0.001 | -0.25 ± 0.67 | **Laser Mach** | <0.001 | 0.191 | 0.006 |
| | 7 days | T3(%) 0.001 | 0.57 ± 0.92 | **Laser Sint** | <0.001 | <0.001 | 1 |
| ***Ti Blast*** | | | | **Al Blast** | 1 | 1 | 0.139 |

| | | | | | | | |
|---|---|---|---|---|---|---|---|
| | 24 hours | T1(*) 0.718 | -0.085 ± 0.69 | **Laser Mach** | 1 | 0.353 | 1 |
| | 3 days | T2(^) 0.298 | -0.19 ± 0.87 | **Laser Sint** | 1 | <0.001 | 0.011 |
| | 7 days | T3($^{\%}$) 0.136 | -0.36 ± 0.64 | **Cor Blast** | <0.001 | 1 | <0.001 |
| ***Laser Sint*** | | | | **Ti Blast** | 1 | <0.001 | 0.011 |
| | 24 hours | T1(*) 0.001 | -0.23 ± 0.62 | **Al Blast** | 1 | <0.001 | <0.001 |
| | 3 days | T2(^) 0.003 | 0.78 ± 0.88 | **Laser Mach** | 1 | 0.354 | 0.204 |
| | 7 days | T3($^{\%}$) <0.001 | 0.32 ± 0.54 | **Cor Blast** | <0.001 | <0.001 | 1 |
| ***Laser Mach*** | | | | **Ti Blast** | 1 | 0.353 | 1 |
| | 24 hours | T1(*) 0.019 | -0.34 ± 0.37 | **Al Blast** | 0.403 | 0.464 | 0.007 |
| | 3 days | T2(^) 0.001 | 0.29 ± 0.94 | **Laser Sint** | 1 | 0.354 | 0.204 |
| | 7 days | T3($^{\%}$) 0.288 | -0.16 ± 0.55 | **Cor Blast** | <0.001 | 0.191 | 0.006 |

**Supplemental Table S3. Summary table of the ADSCs'm-RNA expression on different implants at various time points.** Summary and comparison of the data analysis of the m-RNA (*alp, osn,* and *coll-I*) synthetized by the ADSCs on the different implants at different time points. The data is significant at p≤0.05 (paired t-test). Abbreviations: alkaline phosphatase(*alp*); osteonectin(*osn*); collagen type I (*coll-I*).

| | | ***coll-I*** | | | ***alp*** | | | ***osn*** | | |
|---|---|---|---|---|---|---|---|---|---|---|
| ***Reference Implant*** | **Implant Type** | **24 hours** | **3 days** | **7 days** | **24 hours** | **3 days** | **7 days** | **24 hours** | **3 days** | **7 days** |
| ***Ti Blast*** | Al Blast | <0.001 | 0.038 | <0.001 | <0.001 | <0.001 | <0.001 | N/C | <0.001 | <0.001 |
| | Laser Mach | <0.001 | <0.001 | <0.001 | <0.001 | <0.001 | <0.001 | <0.001 | <0.001 | <0.001 |
| | Laser Sint | <0.001 | <0.001 | <0.001 | <0.001 | <0.001 | <0.001 | <0.001 | <0.001 | <0.001 |
| | Cor Blast | 0.037 | <0.001 | <0.001 | 0.191 | <0.001 | <0.001 | <0.001 | <0.001 | <0.001 |
| ***Al Blast*** | Ti Blast | <0.001 | 0.038 | <0.001 | <0.001 | <0.001 | <0.001 | N/C | <0.001 | <0.001 |
| | Laser Mach | <0.001 | <0.001 | <0.001 | <0.001 | <0.001 | <0.001 | <0.001 | <0.001 | <0.001 |
| | Laser Sint | <0.001 | <0.001 | <0.001 | <0.001 | <0.001 | <0.001 | <0.001 | 0.009 | <0.001 |
| | Cor Blast | <0.001 | <0.001 | <0.001 | <0.001 | <0.001 | <0.001 | <0.001 | 0.107 | <0.001 |
| ***Laser Mach*** | Ti Blast | <0.001 | <0.001 | <0.001 | <0.001 | <0.001 | <0.001 | <0.001 | <0.001 | <0.001 |
| | Al Blast | <0.001 | <0.001 | <0.001 | <0.001 | <0.001 | <0.001 | <0.001 | <0.001 | <0.001 |
| | Laser Sint | 1 | 0.473 | 1 | 0.005 | <0.001 | 0.406 | <0.001 | 0.002 | 0.001 |
| | Cor Blast | <0.001 | 0.001 | <0.001 | <0.001 | <0.001 | <0.001 | 0.001 | <0.001 | <0.001 |
| ***Laser Sint*** | Ti Blast | <0.001 | <0.001 | <0.001 | <0.001 | <0.001 | <0.001 | <0.001 | <0.001 | <0.001 |
| | Al Blast | <0.001 | <0.001 | <0.001 | <0.001 | <0.001 | <0.001 | <0.001 | 0.009 | <0.001 |
| | Laser Mach | 1 | 0.473 | 1 | 0.005 | <0.001 | 0.406 | <0.001 | 0.002 | 0.001 |
| | Cor Blast | <0.001 | 0.07 | <0.001 | <0.001 | 0.51 | <0.001 | <0.001 | 1 | <0.001 |
| ***Cor Blast*** | Ti Blast | 0.037 | <0.001 | <0.001 | 0.191 | <0.001 | <0.001 | <0.001 | <0.001 | <0.001 |
| | Al Blast | <0.001 | <0.001 | <0.001 | <0.001 | <0.001 | <0.001 | <0.001 | 0.107 | <0.001 |
| | Laser Mach | <0.001 | 0.001 | <0.001 | <0.001 | <0.001 | <0.001 | 0.001 | <0.001 | <0.001 |
| | Laser Sint | <0.001 | 0.07 | <0.001 | <0.001 | 0.51 | <0.001 | <0.001 | 1 | <0.001 |

**Supplemental Table S4.** Comparison of m-RNA expression on different implants at various time points. An overview of the data analysis comparison of the m-RNA (*alp*, *osn*, and *coll-I*) expressed by ADSCs on various implants at different time points. The data is significant with *24h-3days, $^{\wedge}$24h-7days and $^{\%}$3-7days (paired t-test) indicating significance. Abbreviations: alkaline phosphatase (*alp*); osteonectin (*osn*); collagen type I (*coll-I*).

| *Implant* | Time points | *coll-I* | *alp* | *osn* |
|---|---|---|---|---|
| ***Ti Blast*** | T1/T2 (*) | 0.78 | 0.909 | 0.002 |
| | T1/T3 (^) | 0.007 | 0.001 | <0.001 |
| | T2/T3 (%) | 0.001 | 0.001 | 0.001 |
| ***Al Blast*** | T1/T2 (*) | 0.017 | N/D | 0.01 |
| | T1/T3 (^) | 0.001 | 0.001 | <0.001 |
| | T2/T3 (%) | 0.003 | 0.001 | <0.001 |
| ***Laser Mach*** | T1/T2 (*) | 0.002 | 0.013 | 0.027 |
| | T1/T3 (^) | 0.004 | 0.004 | 0.018 |
| | T2/T3 (%) | 0.017 | 0.003 | 0.022 |
| ***Laser Sint*** | T1/T2 (*) | 0.006 | 0.686 | 0.001 |
| | T1/T3 (^) | 0.212 | 0.263 | <0.001 |
| | T2/T3 (%) | 0.851 | 0.036 | <0.001 |
| ***Cor Blast*** | T1/T2 (*) | 0.001 | 0.008 | 0.007 |
| | T1/T3 (^) | 0.425 | 0.443 | 0.001 |
| | T2/T3 (%) | 0.007 | 0.002 | 0.001 |
